# Effects of Various Drying Methods on Physicochemical Characteristics and Textural Features of Yellow Croaker (*Larimichthys Polyactis*)

**DOI:** 10.3390/foods9020196

**Published:** 2020-02-15

**Authors:** Bo-Sub Kim, Boung-Jun Oh, Jeung-Hee Lee, Young Seung Yoon, Hae-In Lee

**Affiliations:** Mokpo Marine Food-Industry Research Center, Mokpo 58621, Korea; kbsubi1982@naver.com (B.-S.K.); bjohkim@hanmail.net (B.-J.O.); bluebabyi@nate.com (J.-H.L.); moinet@gmail.com (Y.S.Y.)

**Keywords:** yellow croaker, hot air drying, low temperature vacuum drying, freeze drying, physicochemical characteristics, textural properties

## Abstract

The physicochemical characteristics and textural properties of yellow croaker treated by hot air drying (HAD), low temperature vacuum drying (LVD), and freeze drying (FD) methods were studied. The dried fish by LVD had the lowest moisture content and highest protein. The volatile basic nitrogen values of dried fish by HAD, LVD, and FD were 66.27, 34.38, and 33.03 mg/100 g sample, respectively. The predominant amino acids of dried fish treated by LVD and FD were lysine, taurine, alanine, and glutamic acid, and the predominant ones by HAD were the remaining amino acids analyzed in this study, except lysine, taurine, alanine, and glutamic acid. By using the color parameters, the L* and b* values by LVD showed light brown and yellow colors of the fish. The textural properties of dried fish by LVD were softer and more chewable than those of HAD and FD. In the stereo-micrographs, the flesh of dried fish by LVD compared to others showed minimization of texture damage, resilient tissues, much fish oil, and were light brown in color. Taken together, these results suggest that LVD rather than HAD and FD provide good qualities of dried fish in terms of physicochemical characteristics and textural properties.

## 1. Introduction

The yellow croaker (*Larimichthys polyactis*), a fish belonging to Sciaenidae, is one of benthopelagic warm-temperate fish species. It is widely distributed in the Yellow Sea and East China Sea. The fish spawns in the tideland of Korea from mid-March to June, and is mainly caught in the Yellow Sea of Korea [1].

Due to their huge commercial values, yellow croakers have received substantial attention in Korea and China [2]. Recently, the annual catch of the fish in the East China Sea has reached over 12,000 tons [3]. The fish is favored by consumers looking for its tender texture and abundant nutrition with high-quality protein and unique flavor [3,4]. A lot of yellow croakers and their products are consumed in Korea, Japan, and other countries. The fish can be processed into a variety of food products, such as salted and dried fish, canned fish, grilled fish, etc. [5]. In Korea, more than 90% of yellow croakers consumed are processed as salted and dried fish.

Fish are important substances in global food markets. They contain omega-3 fatty acids, high-quality protein, essential amino acids, minerals, vitamins, and trace elements, etc. Thus, the fish enhance nutrition and are good for health [6]. It is reported that eicosapentaenoic acid (EPA) and docosahexaenoic acid (DHA) have the ability to lower blood pressure and heart rate by improving blood vessel function [7]. Thus, omega-3 fatty acids may reduce the risk of cardiovascular disease and hypertension by reducing cholesterol levels and triglycerides in the blood [7].

As dried yellow croaker is a fatty and high-protein food, it is easily decomposed during natural drying [8]. Thus, it is important that a drying method can increase storage of dried fish. A traditional method for dried fish is solar drying and is a low-priced method. However, solar drying has critical shortcomings in that yellow croakers can be frequently contaminated by dusts, rats, and unhygienic insects, etc. In addition, solar drying is mainly affected by climate conditions [9]. In the past, when refrigeration and cold storage technology were not developed, the fish had to be processed into a saltier and drier state in order to increase storage.

Nowadays, due to the concern of various diseases caused by high salinity and the influence of yellow and fine dusts, we need to use a hygienic and safe drying method rather than the traditional one [1]. As this trend continues, drying techniques for low-sodium seafoods are being developed [1,10]. Recently, hot-air drying (HAD) methods have been widely used. However, this method has negative influences on the final quality of fish products [9]. It causes shrinkage, discoloration, and loss of heat-sensitive ingredients, etc., of the products. In addition, it takes generally a long time to dry, and consumes a lot of energy [9]. On the other hand, low temperature vacuum drying (LVD) and freeze drying (FD) methods can preserve the heat-sensitive ingredients of foods [11]. By LVD and FD methods, shrinkage of food is alleviated, and flavor, aroma, and vitamins can be preserved to a good condition [12]. 

There are several studies on the yellow croakers dried by HAD and vacuum drying [7,13]. However, studies on physicochemical properties of dried fish especially by LVD and FD methods have not been reported yet. The purpose of this study is to examine the physicochemical properties of dried fish by various methods, and to select a suitable drying method for freshness and good quality of dried fish.

## 2. Materials and Methods 

### 2.1. Sample Preparation for Drying

Yellow croakers were purchased from Daechungsusan Food Company in Mokpo City, Korea. The weight and length of fish ranged from 80 to 90 g, and from 19 to 21 cm, respectively. The frozen fish were thawed overnight in a storage room at 4 °C before the salting process. The solution of 10% bay salt was used for salting at 17 °C for 3 h. Then the samples were shortly rinsed with fresh water and kept in a cold air drying room at 17 °C for 24 h for removing moisture from the samples. The fish prepared via the above process were used as the control in this study, and used for drying experiments.

### 2.2. Drying Experiments

The drying techniques and drying equipment were provided by the Mokpo marine food-industry research center. Yellow croakers were dried by low temperature vacuum drying, freeze drying, and hot air drying methods. All tests were conducted in triplicate. The conditions for the drying tests were as follows: 

The vacuum drying experiments were carried out in a low temperature vacuum dryer (Korea bio solution Co., Busan, Korea) [10]. The heated wall temperatures were in the range 50–60 °C. A vacuum pump was attached to the drying chamber to lower the pressure. The vacuum levels were set at −720 mmHg. The drying time to reduce moisture content to 35%–50% was 20 h. The fish sample was frozen at −60 °C for 120 min. After, freeze drying was carried out in a freeze dryer (Operon Co., Kimpo, Korea) for 48 h at a temperature of −40 °C with a vacuum of −750 mmHg. The final moisture content of dried hydrolysates was less than 50%. The hot air drying was carried out in a hot air mechanical dryer (J-ITEC Co., Ansan, Korea) at controlled temperatures of 60 °C with an air velocity of 2.0 ± 0.3 m/s maintained [1]. The drying time to reduce moisture content to 35%–50% was 96 h. All samples were dehydrated until they reached the final moisture content (30%–50%). Dried fish samples were packed tightly in polyethylene bags and stored at −20 °C until analysis for the experiment. And then, obtained fillets from the dried fish before experiment and was used for analyses.

### 2.3. Analysis of Proximate Composition

The proximate composition such as moisture, crude protein, crude lipid, and ash content were analyzed according to standard procedure given in Association of Official Analytical Chemists (AOAC) methods [14]. The moisture content of samples was determined by weight loss after 12 h of drying at 105 °C in the oven (WOF-155, DAIHAN, Seoul, Korea). The lipid content was determined by the Soxhlet method with a solvent extraction system (Soxtec Avanti SE-416, Gerhardt, Germany). The protein content was determined by the Kjeldahl method with the automatic nitrogen analyzer (VAPODEST 50S, Gerhardt, Germany). The ash content was determined according to the AOAC.

### 2.4. Measurement of Volatile Basic Nitrogen

The volatile basic nitrogen (VBN) of samples was evaluated according to the Conway micro-diffusion method [15]. Briefly, 10 g of the samples was homogenized in 45 mL 5% trichloroacetic acid solution at 14,320× *g* for 1 min and filtered with Whatman No. 1 filter paper. A total of 1 mL of the filtrated sample was transferred to the outer place of Conway chamber. Then, 1 mL of 0.01 N boric acid (H_3_BO_3_) solution was added to the inner place of the Conway chamber. The Conway unit was closed immediately after the addition of 1 mL of K_2_CO_3_ solution was added to the outer place. After incubating at 37 °C for 1 h, titration was progressed with 0.02 N sulfuric acid to the inner place of the chamber. The measurement data were expressed as mg/100 g sample.

### 2.5. Amino Acid Analysis

For amino acid analysis, the dilution of samples was mixed with the same volume of 5% (*w/v*) trichloroacetic acid and centrifuged for 15 min at 12,000× *g* at 4 °C to remove proteins. The supernatant was collected and filtered through the 0.2 μm syringe filter (ADVANTEC Co., Tokyo, Japan). Then, the prepared sample was separated by cation-exchange chromatography and identified spectrophotometrically after the reaction with the ninhydrin reagent. Eighteen basic amino acids (alanine, arginine, asparagine, citrulline, glutamic acid, glycine, histidine, leucine, lysine, methionine, ornithine, phenylalanine, proline, serine, taurine, threonine, tyrosine, and valine) were assayed using the automated amino acid analyzer (L-8900, Hitachi, Tokyo, Japan).

### 2.6. Color Measurements

The color parameters of samples were evaluated using the formula CIE-L*a*b*, where L* indicates lightness, a* redness/greenness, and b* yellowness/blueness [16,17]. The instrumental color measurement of samples was carried out using the Minolta photocolorimeter (CR-400, Osaka, Japan).

### 2.7. Textural and Morphological Characters

The textural properties of samples were analyzed using the texture analyzer (TMS-Pro, Food Technology Corporation, Germany). Cylindrical samples (30 mm diameter) were cut from loaf slices with a thickness of 15 mm, and compressed to a 10 mm depth at a crosshead speed of 60 mm/min. The texture profile analysis (TPA) was carried out at room temperature (25 °C) and the analysis was completed within 60 s. A force-time graph was generated and textural parameters, such as hardness, cohesiveness, adhesiveness, springiness, gumminess, and chewiness, were calculated by the software provided along with the instrument. The important morphological characters were photographed with the automated stereo-microscope (Leica M205A stereomicroscope, Wetzlar, Germany).

### 2.8. Statistical Analysis

Three measurements were made for each sample in the same group and average value was reported for each parameter. All data are presented as the means ± standard error (SE). Statistically significant variation among the groups was determined by one-way analysis of variance (ANOVA) by using SPSS software (Chicago, IL, USA). The differences between the control group and other group were compared by the Student’s *t*-test, respectively. For dried yellow croaker groups (HAD, LVD, FD), significant differences between means were identified by one-way ANOVA followed by Duncan’s multiple-range test. A *p* < 0.05 was considered to indicate significance.

## 3. Results 

### 3.1. Proximate Composition 

Higher moisture content will make food products susceptible to enzymatic and microbial decomposition [13]. Particularly, fish products with a higher moisture level are susceptible to spoilage if not conserved properly. 

The moisture content of the yellow croakers dried with the various methods ranged from 38.56% to 47.38% on a fresh matter basis (Table 1). The moisture content of dried fish by LVD was the lowest as 38.56%. Additionally, the moisture content by HAD and FD were 47.08% and 47.38%, respectively (Table 1). The crude lipid of dried fish by LVD was the lowest as 12.56% on a dry matter basis. In addition, the crude lipid by FD and HAD were 13.43% and 14.67%, respectively. The crude protein of dried fish by LVD was the highest as 41.48% on a dry matter basis. Additionally, the crude protein by FD and HAD were 33.54% and 31.32%, respectively. The crude ash of dried fish by LVD was the highest as 6.26% on a dry matter basis. Finally, the crude protein by FD and HAD were 4.02% and 3.74%, respectively.

### 3.2. Total VBN Value

Many researchers in fish and seafood industries use the VBN as a parameter to determine the quality of fish and seafood. VBN is produced by deterioration of fish and regarded as a noxious smell [18]. Thus, VBN value has been used as a freshness factor of fish in fisheries [19]. 

**Figure 1:**
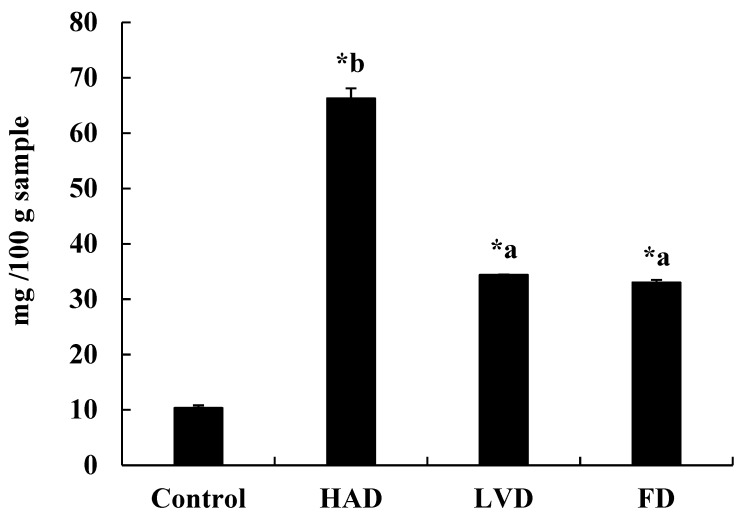
Analysis of volatile basic nitrogen (VBN) in the yellow croakers by various drying methods. Values are mean ± SE. The values are significantly different between groups according to Student’s *t*-test: * *p* < 0.05, Control vs. other groups (HAD, LVD, FD), respectively. The values not sharing a common letter (^a^ and ^b^) are significantly different among groups using one-way ANOVA followed by Duncan’s multiple-range test at *p* < 0.05. Control, yellow croakers by cold air drying to remove moisture. HAD, yellow croakers by hot air drying. LVD, yellow croakers by low temperature vacuum drying. FD, yellow croakers by freeze drying.

The VBN values of fish dried by various methods are shown in Figure 1. The VBN value of dried fish by HAD, LVD, and FD were 66.27, 34.38, and 33.03 mg/100 g sample, respectively. The VBN value by HAD was significantly higher than that of LVD and FD.

### 3.3. Free Amino Acid Composition

Amino acids are the main factors that have been used to evaluate the nutritional value of fish and aquatic organisms, etc. [20]. The amino acids are classified as essential and non-essential. The essential amino acids in this study were arginine, histidine, isoleucine, leucine, lysine, methionine, phenylalanine, threonine, and valine. The non-essential amino acids were alanine, asparagine, citrulline, glutamic acid, glycine, ornithine, proline, serine, taurine, and tyrosine. 

The free amino acid level of dried fish by various methods was higher than that of the control, except asparagine by HAD (Table 2). The dried fish by LVD showed the highest content of lysine (96.82 mg/100 g) and taurine (88.82 mg/100 g) compared to that by HAD and FD. The dried fish by FD compared to others showed the highest content of asparagine (17.13 mg/100 g) and serine (15.97 mg/100 g). The dried fish by HAD showed the highest content of alanine, leucine, glutamic acid, citrulline, proline, valine, glycine, phenylalanine, methionine, threonine, tyrosine, histidine, and ornithine as compared to other methods. Additionally, the total free amino acids by HAD, LVD, and FD were 1141.49, 674.7, and 403.06 mg/100 g, respectively.

### 3.4. Color Values

The color of foods is generally determined in the L*a*b*, which is an international standard color parameter [21]. L* value indicates lightness component in an image. +L* value shows white in color, and −L* value shows black in color. Therefore, L* values are usually employed to indicate the browning of foods during processing. +a* value of an image shows red in color, while −a* value shows green. +b* value of an image shows yellow in color, while −b* value shows blue. 

The L* values of dried fish by LVD were the highest as 45.85 (Table 3). Additionally, the L* values by FD and HAD were 39.79 and 38.82, respectively. Thus, the L* values by LVD compared to others showed a brighter color of the fish. The a* values of dried fish by HAD, LVD, and FD were 2.34, 2.68, and 2.69, respectively, and not significantly different among methods. The b* values of dried fish by LVD was the lowest as 9.91. Additionally, the b* values by FD and HAD were 10.65 and 12.44, respectively. Thus, the b* values by LVD compared to others showed less yellow in the color of the fish. 

### 3.5. Textural Properties and Stereo-Micrographs

**Figure 2:**
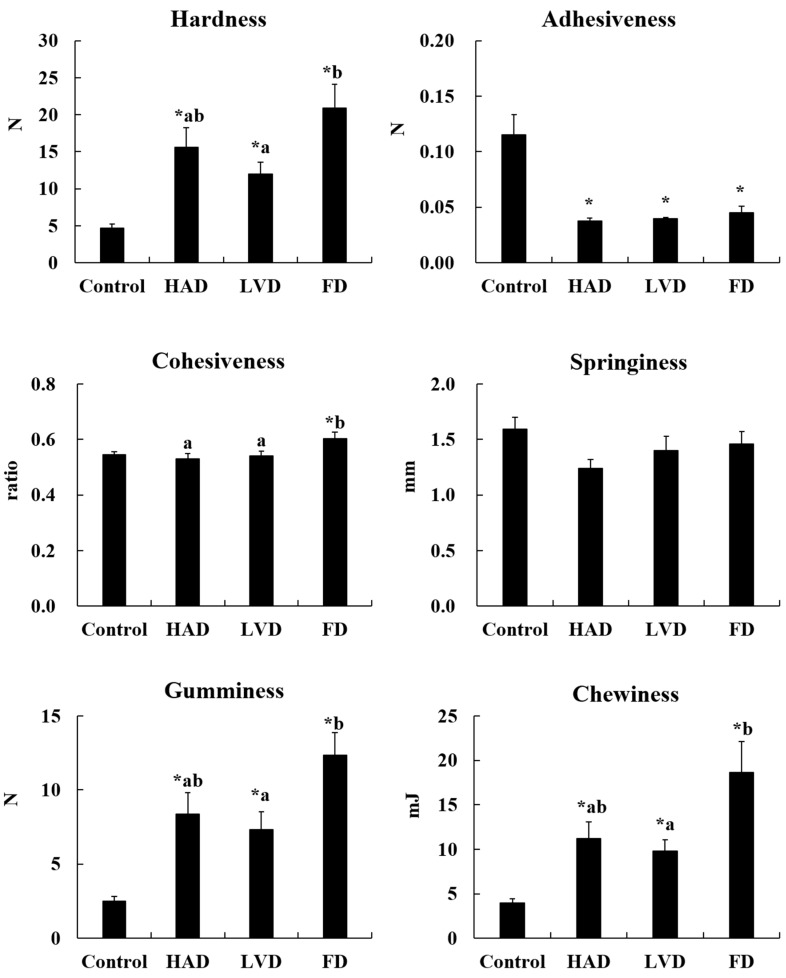
Analysis of textural properties in the yellow croakers by various drying methods. Values are mean ± SE. The values are significantly various between groups according to Student’s *t*-test: * *p* < 0.05, Control vs. other groups (HAD, LVD, FD), respectively. The values not sharing a common letter (^a^ and ^b^) are significantly different among groups using one-way ANOVA followed by Duncan’s multiple-range test at *p* < 0.05. Control, yellow croakers by cold air drying to remove moisture. HAD, yellow croakers by hot air drying. LVD, yellow croakers by low temperature vacuum drying. FD, yellow croakers by freeze drying.

Texture is one of the crucial indexes of food quality. Texture profile analysis (TPA) is executed to assess the textural properties of food. In order to examine the texture profiles of dried fish, hardness, adhesiveness, cohesiveness, chewiness, gumminess, and springiness were analyzed (Figure 2). The hardness values of dried fish by HAD, LVD, and FD were 15.61, 11.96, and 18.63 N, respectively. The FD fish were harder than others, and the LVD fish were softer. The chewiness values by HAD, LVD, and FD were 11.17, 9.78, and 18.63 mJ, respectively. Cohesiveness and gumminess in the FD fish group was significantly higher relative to the HAD and LVD groups. The LVD fish were more chewable than others, and the FD ones were less chewable. The springiness and adhesiveness values were not significantly different among drying methods. Taken together, hardness and chewiness values by HAD, LVD, and FD were significantly higher than those of the control. The LVD samples were softer and more chewable than the other ones. 

**Figure 3:**
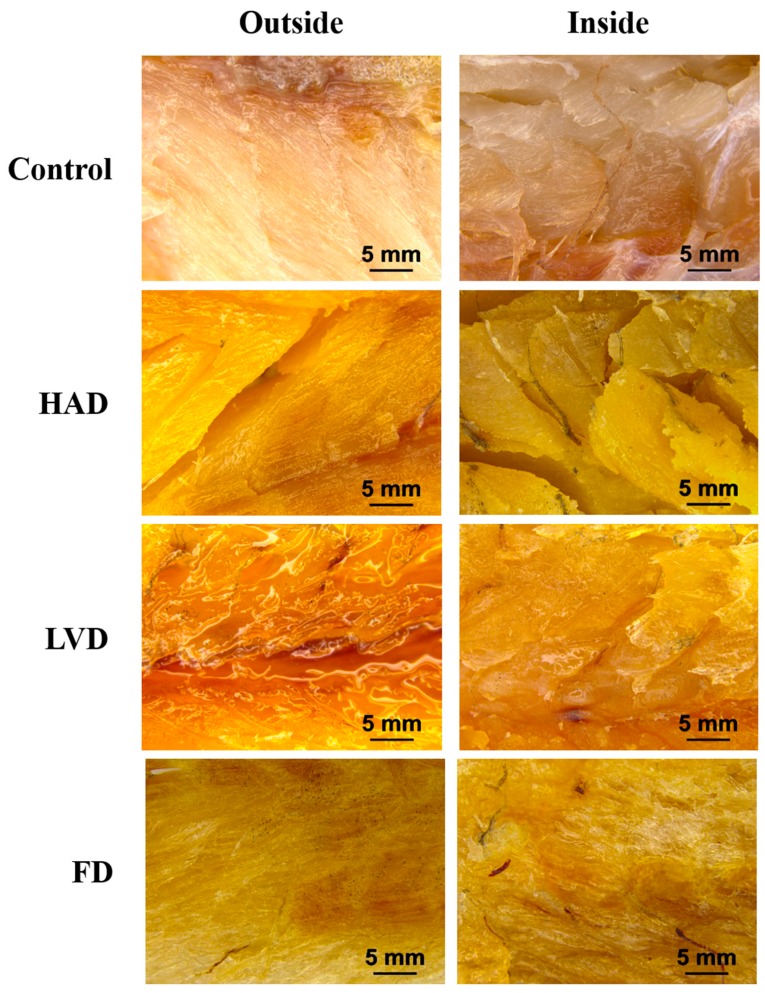
Outside (left) and inside (right) longitudinal section of stereo-micrographs in the yellow croakers by various drying methods. Scale bars: 5 mm. Control, yellow croakers by cold air drying to remove moisture. HAD, yellow croakers by hot air drying. LVD, yellow croakers by low temperature vacuum drying. FD, yellow croakers by freeze drying.

The stereo-micrographs of dried fish by various methods are shown in Figure 3. The flesh of dried fish by LVD compared to others showed resilient tissues, much fish oil, and were light brown in color. However, the flesh of dried fish by HAD and FD showed disappearance of fish oil and were dark brown in color. In addition, in the fish flesh by HAD severely broken tissues were observed.

## 4. Discussion

Dried fish are popular processed foods in many countries of the world. They are generally produced by diverse drying methods, which play important roles in the physicochemical and sensory properties of fish products [22]. 

Moisture content was the primary factor affecting the quality and storage period of dried fish. [23]. It was reported that microbial contamination during processing and storage depended on the moisture content of dried fish [24]. If the moisture content of dried fish is high, then deteriorative changes may result in browning, and rancid and fungal contamination of fish products [25]. The fish with a moisture content of 80% or more were vulnerable to spoilage if they are not properly stored [13].

The moisture content of dried fish distributed from fish markets was reported from 36.1% to 52.0% [26]. It was also reported that the moisture content of 35% to 40% in dried yellow corvenia was the optimum level for preventing surface hardening [1]. In the present study, the moisture content of dried yellow croakers by LVD is 38.56%, and the one by HAD and FD is 47.08% and 47.38%, respectively (Table 1). This result suggests that the LVD rather than others could be better for the longer storage of dried fish by prevention of microbial contamination.

Protein content in dried fish was increased due to the dehydration of water molecules that exist between proteins [27]. This study also showed that the dried fish by LVD compared to those by HAD and FD had the highest protein content and the lowest moisture content (Table 1). This study showed a positive correlation between protein and lipid contents (r = 0.732, *p* < 0.01) of the dried fish.

VBN is the total amount of volatile nitrogen bases together with the nitrogen that is caused by microbial activities [28]. A number of VBN components, such as dimethylamine, trimethylamine, and ammonia, are released from the fish decomposed by bacteria [22]. The VBN in seafood is generally used as a spoilage indicator [29]. The VBN level in dried fish acceptable for human ingestion is below 50 mg/100 g sample [30]. Above that level, fish products are considered as undesirable. In this study, the VBN levels by LVD and FD were below 35 mg % (Figure 1). However, the VBN levels by HAD were above 65 mg/100 g sample, resulting in an obnoxious smell not suitable for ingestion. It is well known that dried fish are easily decomposed even at room temperature during storage. HAD methods have been commonly used to dry fish, but high temperature causes negative effects on fish quality [9]. The present result also demonstrated that dried fish by HAD performed at 60 °C were poor quality. The fish by low temperature drying such as LVD (30–35 °C inside vacuum chamber) and FD (–40 °C) compared to HAD showed lower VBN levels and better quality. This result suggests that LVD and FD are the recommended method for freshness and good quality of dried fish.

Amino acid profile is used to evaluate the nutritive quality of fish and aquatic organisms [31]. Free amino acids were reported to be abundant in seafood, and generate characteristics of fish flavor [32]. In this study, the amino acid profile by HAD is quite different from that by LVD and FD (Table 2). The predominant amino acids in dried fish by LVD and FD were lysine, taurine, alanine, and glutamic acid. Vice versa, the ones by HAD were the remaining essential amino acids except lysine, taurine, alanine, and glutamic acid. This result suggests that the HAD method by heat and the LVD and FD methods by low temperature, respectively, may have different influences on dried fish, resulting in production of different amino acid profiles.

Color is one of the factors that determine good processed marine products [33]. The change of fish color was caused by unfavorable environments [16]. Moreover, the oxidation of lipids is an important factor that leads to browning of dried fish-muscles by interacting with proteins [34]. It was reported that as processing temperature and time was increased or moisture level was decreased, darker brown fish were produced [17]. 

The species of yellow croakers has a grey and light yellow in the body and belly, which is very important for the quality evaluation of consumers [35]. Thus, the development of drying techniques to inhibit browning is one important strategy for quality of dried fish production. In this study, all drying methods may affect color parameters of dried fish (Table 3). By the evaluation of L* and b* values, respectively, the dried fish by LVD compared to FD and HAD were brighter and less yellow in color. Additionally, in the stereo-micrograph observation, the LVD rather than other methods showed light brown in color in the flesh of dried fish (Figure 3). This result suggests that the LVD method produces a light brown color of dried yellow croakers compared to the other methods.

It is reported that drying tends to increase tissue hardness [36]. Hardness is one of the factors that determines the acceptability of foods by consumer [37]. Chewiness is a criterion of energy required to masticate foods [38]. High chewiness value means that it takes longer time to breakdown foods during chewing [20]. In this study, the values of hardness and chewiness of dried fish by LVD were softer and more chewable than by the others, respectively. Additionally, there is a high correlation (r = 0.949) between the hardness and the chewiness values of fish by the drying methods. This result suggested that the dried fish by LVD were found to be acceptable for the consumer. 

In the present study, our results suggest that the dried yellow croakers by LVD rather than HAD and FD present relatively better quality in terms of physicochemical characteristics and textural properties. Thus, the LVD compared to the HAD and FD is an alternative and potential technique for drying of various fish species.

## Figures and Tables

**Table 1:** Analysis of moisture content, crude lipid, crude protein, and crude ash in the yellow croakers by various drying methods ^1.^

	Control ^2^	HAD ^3^	LVD ^4^	FD ^5^
Moisture content (%)	71.29 ± 0.07	47.08 ± 0.31 *^,b^	38.56 ± 0.14 *^,a^	47.38 ± 0.25 *^,b^
Crude lipid (%)	7.25 ± 0.20	14.67 ± 0.13 *^,c^	12.56 ± 0.08 *^,a^	13.43 ± 0.18 *^,b^
Crude protein (%)	19.03 ± 0.14	31.32 ± 0.25 *^,a^	41.48 ± 0.16 *^,c^	33.54 ± 0.34 *^,b^
Crude ash (%)	2.23 ± 0.07	3.74 ± 0.08 *^,a^	6.26 ± 0.12 *^,b^	4.02 ± 0.03 *^,a^

^1^ The values are significantly different between groups according to Student’s *t*-test: * *p* < 0.05, Control vs. other groups (HAD, LVD, FD), respectively. The values not sharing a common letter (^a^ and ^b^) are significantly different among groups using one-way ANOVA followed by Duncan’s multiple-range test at *p* < 0.05. ^2^ Control, yellow croakers by cold air drying to remove moisture. ^3^ HAD, yellow croakers by hot air drying. ^4^ LVD, yellow croakers by low temperature vacuum drying. ^5^ FD, yellow croakers by freeze drying.

**Table 2:** Analysis of 18 free amino acids in the yellow croakers by various drying methods (mg/100 g) ^1.^

	Control ^2^	HAD ^3^	LVD ^4^	FD ^5^
**Essential amino acid**				
Arginine	3.86	-	-	-
Histidine	6.12	24.02	15.18	7.61
Isoleucine	4.95	51.06	22.66	13
Leucine	8.99	115.18	44.01	23.19
Lysine	43.48	80.09	96.82	52.52
Methionine	4.35	50.51	19.14	9.39
Phenylalanine	4.71	51.41	21.69	10.37
Threonine	9.37	36.66	21.17	18.56
Valine	7.63	75.51	32.04	19.05
**Non-essential amino acid**				
Alanine	17.96	157.92	76.26	39.56
Asparagine	9.23	9.06	12.68	17.13
Citrulline	14.93	100.04	47.25	25.89
Glutamic acid	15.71	114.38	64.3	32.68
Glycine	8.02	74.15	36.24	13.73
Ornithine	5.75	17.67	14.53	6.75
Proline	6.93	87.23	33.2	21.6
Serine	8.59	10.03	13.45	15.97
Taurine	45.54	50.9	88.82	70.19
Tyrosine	3.72	35.67	15.26	5.87
**Total**	**229.84**	**1141.49**	**674.7**	**403.06**

^1^ Values are mean ± SE. ^2^ Control, yellow croakers by cold air drying to remove moisture. ^3^ HAD, yellow croakers by hot air drying. ^4^ LVD, yellow croakers by low temperature vacuum drying. ^5^ FD, yellow croakers by freeze drying.

**Table 3:** Analysis of color parameters in the yellow croakers by various drying methods ^1.^

	Control ^2^	HAD ^3^	LVD ^4^	FD ^5^
L*	51.60 ± 1.04	38.82 ± 1.67 *^,a^	45.85 ± 0.89 *^,b^	39.79 ± 1.16 *^,a^
a*	−0.82 ± 0.24	2.34 ± 0.49 *	2.68 ± 0.31 *	2.69 ± 0.09 *
b*	6.08 ± 0.16	12.44 ± 0.46 *^,b^	9.91 ± 0.52 *^,a^	10.65 ± 0.38 *^,a^

^1^ The values are significantly different between groups according to Student’s *t*-test: * *p* < 0.05, Control vs. other groups (HAD, LVD, FD), respectively. The values not sharing a common letter (^a^ and ^b^) are significantly different among groups using one-way ANOVA followed by Duncan’s multiple-range test at *p* < 0.05. ^2^ Control, yellow croakers by cold air drying to remove moisture. ^3^ HAD, yellow croakers by hot air drying. ^4^ LVD, yellow croakers by low temperature vacuum drying. ^5^ FD, yellow croakers by freeze drying.
